# Poly[ADP-Ribose] Polymerase-1 Expression Is Related To Cold Ischemia, Acute Tubular Necrosis, and Delayed Renal Function In Kidney Transplantation

**DOI:** 10.1371/journal.pone.0007138

**Published:** 2009-09-28

**Authors:** Francisco O'Valle, Raimundo G. M. Del Moral, María del Carmén Benítez, David Martín-Oliva, Mercedes Gómez-Morales, David Aguilar, José Aneiros-Fernández, Pedro Hernández-Cortés, Antonio Osuna, Francesc Moreso, Daniel Serón, Francisco J. Oliver, Raimundo G. Del Moral

**Affiliations:** 1 Department of Pathology and Institute of Biopathology and Regenerative Medicine (IBIMER), School of Medicine, University of Granada, Granada, Spain; 2 Department of cell Biology, University of Granada, Granada, Spain; 3 Department of Traumatology and Orthopaedic Surgery, San cecilio University Hospital, Granada, Spain; 4 Department of Nephrology, Virgen de las Nieves University Hospital, Granada, Spain; 5 Department of Nephrology, Bellvitge University Hospital, Barcelona, Spain; 6 Institute of Parasitology and Biomedicine, CSIC, Armilla, Granada, Spain; L' Istituto di Biomedicina ed Immunologia Molecolare, Consiglio Nazionale delle Ricerche, Italy

## Abstract

Cold ischemia time especially impacts on outcomes of expanded-criteria donor (ECD) transplantation. Ischemia-reperfusion (IR) injury produces excessive poly[ADP-Ribose] Polymerase-1 (PARP-1) activation. The present study explored the hypothesis that increased tubular expression of PARP-1 contributes to delayed renal function in suboptimal ECD kidney allografts and in non-ECD allografts that develop posttransplant acute tubular necrosis (ATN).

**Materials and Methods:**

Nuclear PARP-1 immunohistochemical expression was studied in 326 paraffin-embedded renal allograft biopsies (193 with different degrees of ATN and 133 controls) and in murine Parp-1 knockout model of IR injury.

**Results:**

PARP-1 expression showed a significant relationship with cold ischemia time (r coefficient = 0.603), time to effective diuresis (r = 0.770), serum creatinine levels at biopsy (r = 0.649), and degree of ATN (r = 0.810) (p = 0.001, Pearson test). In the murine IR model, western blot showed an increase in PARP-1 that was blocked by Parp-1 inhibitor. Immunohistochemical study of PARP-1 in kidney allograft biopsies would allow early detection of possible delayed renal function, and the administration of PARP-1 inhibitors may offer a therapeutic option to reduce damage from IR in donor kidneys by preventing or minimizing ATN. In summary, these results suggest a pivotal role for PARP-1 in the ATN of renal transplantation. We propose the immunohistochemical assessment of PARP-1 in kidney allograft biopsies for early detection of a possible delayed renal function.

## Introduction

Renal ischemia produced during transplantation or otherwise is a major cause of acute kidney injury, initiating a complex and interrelated sequence of events that result in the injury and eventual death of renal cells [1], [2]. Prolonged cold ischemia contributes to organ damage and increases patient morbidity and mortality. Salahudeen et al. recently studied 6,465 kidney transplant patients using United Network for Organ Sharing (UNOS) data and concluded that prolonged cold ischemia is a strong risk factor for delayed graft function and a significant predictor of short-term [3] and long-term [4] graft loss, as reported by other authors [5], [6]. Nevertheless, the pathogenic mechanism has yet to be fully elucidated.

Johnston et al. concluded that cold ischemia time has a major impact on the outcome of transplants from aged and expanded-criteria donors (ECDs). In non-ECD kidneys, very prolonged cold ischemia time is associated with an increase in primary non-function. ECD kidneys from older donors show a greater increase in delayed graft function with longer cold ischemia time. Thus, ECD grafts with cold ischemia time of >8 h have higher delayed graft function rates than do non-ECD grafts with cold ischemia time of >37 h [7].

Early renal transplant dysfunction is mainly caused by ischemic damage (acute tubular necrosis [ATN]), rejection, infection, or cyclosporin A toxicity [8]. The prognosis is complicated by the fact that reperfusion, although essential for the survival of ischemic renal tissue, causes additional damage (reperfusion injury) [9], [10] that contributes to the renal dysfunction and injury associated with ischemia/reperfusion (IR) of the kidney [1], [10].

Poly[ADP-Ribose] Polymerase-1 (PARP-1) (E.C. 2.4.2.30) is a nuclear zinc-finger DNA-binding protein with a molecular weight of 113 kDa that specifically detects DNA-strand breaks or nicks produced by different genotoxic agents in mammalian cells [11]. PARP-1 catalyzes the ADP ribosylation of proteins using NAD(+) as substrate [12]. PARP activation is a consequence of ischemic injury and results in a depletion of intracellular NAD(+) [13], which can only be replenished *via* a reaction that consumes ATP. DNA damage produced by IR injury requires cells to consume large amounts of ATP to support poly(ADP-ribosyl)ation. For this reason, whereas moderate PARP activity protects cellular genome integrity, excessive PARP activation can lead to cell death from ATP depletion [14]–[16]. Our group previously demonstrated that PARP1 expression in tubules of aged donors correlates with functional reserve parameters (serum creatinine and time required to achieve effective diuresis) [17].

The present study was designed to produce clinicopathological evidence to test the hypothesis that increased tubular expression of PARP-1 in human allograft kidneys that are suboptimal or develop ATN posttransplant might be one of the predictive factors for a subsequent delay in renal function.

## Materials and Methods

We studied 326 paraffin-embedded renal allograft biopsies distributed in four groups: two ECD groups, one with and one without the presence of ATN; and two non-ECD groups, one with and one without the presence of ATN. ECDs were selected as specified in UNOS policies and procedures as either ≥60 yrs of age or between 50 and 59 yrs of age with at least two of the following three risk factors: a history of hypertension, a history of cerebrovascular disease, and serum creatinine at any time ≥1.5 mg/dL) [7].

For the ECDs, 220 kidney wedge biopsies were fixed in Glyofix (Pacisa-Giralt, Barcelona, Spain) and embedded in paraffin by microwave-accelerated technique to determine renal lesions at 0-h pre-transplant. Out of these 220 ECD kidneys, 95 biopsies with some degree of ATN and 65 without ATN (serving as control group) were selected for the study. The remaining 60 kidneys were not transplanted due to severe vascular or glomerulo-interstitial renal lesions, and 20 of these were maintained as whole perfused kidneys at 4°C for 48 h in Wisconsin preservation solution. For the non-ECDs, 98 kidney cylinder biopsies were taken between days 5 and 11 post-transplant from oligoanuric recipients, fixed in 10% buffered formalin, and embedded in paraffin by standard procedure to identify ATN and classify its degree as: mild (1) [<10% of tubules with necrotic cells], moderate (2) [10 to 49%], or severe (3) [≥50%]. A further 68 kidney sections (transplant protocol biopsies) from non-ECDs with stable renal function and without morphological evidence of ATN served as a control group. The study was conducted according to the Helsinki declaration and approved by the Ethics Committee of the hospital. All biopsies were taken after written informed consent was obtained and subjects were included in this investigation after agreeing to participate and signing the appropriate consent form.

Nuclear expression of PARP-1 was characterized by incubating sections for 60 min at room temperature with PARP-1 monoclonal antibody (clone A6.4.12) (LabVision Fremont, CA, USA). The immunochemistry study was done on an automatic immunostainer (model autostainer*480*, LabVision) using the polymer peroxidase-based method followed by development with diaminobenzidine (Master Diagnóstica, Granada, Spain). The positivity of immunostaining was calculated semiquantitatively on a 4-point scale (0, absence; 1 [1–9% of tubular nuclei positive]; 2 [10–49%]; 3 [≥50%]). Renal sections incubated with isotype antibody and tonsil sections were used as negative and positive controls, respectively.

Data were gathered on renal function parameters (serum creatinine [mg/dL]), creatinine clearance [mL/min./1.73 m^2^]), donor and recipient age and sex, cold-ischemia time, reanastomosis time, time to effective diuresis (defining effective diuresis in terms of Cockcroft-Gault calculated creatinine clearance rather than need for posttransplantation dialysis), immunosuppression regimens, and number of hemodialysis sessions.

### Ischemia-reperfusion (IR) mouse model

We used 20 male Parp1^+/+^ wild-type and 20 male Parp1^−/−^ knockout C57BL/6 mice (24 wks old and 20–30 g). Knockout mice were obtained according to a previously reported procedure [18]. The mice were kept under stable conditions at the Institute of Parasitology and Biomedicine in Granada with *ad libitum* access to food and water. All experiments were performed according to European Union and Spanish Government guidelines for the ethical care of animals (EU Directive 86/609, RD 223/1988).

Mice were anesthetized by intraperitoneal inoculation of equitensin (2 IU/20 g) and maintained at 37°C on a thermal plate. The left kidney was accessed by anterolateral abdominal horizontal incision of 1.5 cm, and the vascular pedicle was clamped with a model 2A S&T metallic clip (S&T Microlab AG, Rheinfall, Switzerland), maintaining the kidneys within the abdominal cavity under University of Wisconsin solution flow at 4°C. After 45 min of clamping, the clip was removed and the peritoneum and skin were sutured. After 6 and 48 h of reperfusion, the animals were killed with an overdose of sodium pentothal. There were no deaths during postoperative or reperfusion periods.

### Administration of PARP inhibitor to mice

PARP-1 inhibitor 3-aminobenzamide (3-ABA) was purchased from Sigma Chemicals (St Louis, MO) and dissolved in saline at a concentration of 5 mg/mL 3-ABA (10 mg/kg) was administered intraperitoneally at 1 h before ischemic injury. Vehicle-treated mice received the saline injection without 3-ABA. In preliminary control experiments, it was determined that administration of 3-ABA to sham-operated mice had no morphological effect.

### Renal samples and processing

Two groups of kidneys from C57BL/6 Parp1^+/+^ mice and two groups from C57BL/6 Parp1^−/−^ mice were formed, divided between kidneys with 6 h or 48 h of reperfusion (n = 10 for each time and mouse type) and kidneys with 6 h of reperfusion plus PARP-1 inhibitor 3-ABA (n = 10 for each group); two groups of control kidneys for each reperfusion time were also studied (n = 20 for each group). In all animals, the left kidney was subjected to ischemia-reperfusion by clamping the complete renal vascular pedicle, using the right kidney as control. After extraction, each kidney sample was divided longitudinally into two halves. One half, with separated cortex and medulla, was rapidly frozen in isopentane at −50°C and submerged in liquid nitrogen for 10 s to develop western-blotting. The other half was immediately fixed in 10% buffered formalin for 24 h and then paraffin-embedded for morphological study using hematoxylin-eosin and PAS staining.

### Western blot analysis

Tissues extracted from the human and mouse kidney cortex samples were washed with PBS and resuspended in 100 µl lysis buffer (50 mM Tris-HCl pH 8, 0.1 mM EDTA, 0.5% Triton X-100, 12.5 mM β-mercaptoethanol) for 30 min on ice. Pellet was eliminated and sample buffer (50 mM Tris-HCl pH 6.8, 6 M urea, 6% β-mercaptoethanol, 3% SDS, 0.003% bromophenol blue) was added to the supernatant. Proteins were resolved on SDS- 12% polyacrylamide gels and transferred onto Immun-Blot PVDF Membrane (Bio-Rad, Laboratories Irvine, CA, USA). The blot was blocked with 5% milk powder in PBS with 0.1% Tween-20 for 30 min, washed with PBS/Tween, and incubated overnight with anti-poly[ADPribose] (PAR) (TREVIGEN, Gaithersburg, MD), anti-PARP1 (clone C2-10) (Alexis, San Diego, CA, USA), and anti-α-tubulin (Sigma, St Louis MO, USA) antibodies and for 2 h with appropriate secondary antibodies. Bands were visualized by ECL-PLUS (Amersham Biosciences, Piscataways, NJ, USA), and photographs were taken using the ChemiDoc XRS imaging system (Bio-Rad). Positive control: Poly-ADP-ribosylated-PARP protein control for Western Blot (TREVIGEN, Gaithersburg, MD).

### Statistical analysis

The Kolmogorov-Smirnov test was used to assess the normality of the variables. A descriptive analysis was performed, and the Student's *t*-test, one way ANOVA with *post hoc* Bonferroni test, chi square test, and Pearson's correlation were applied to determine statistical significances. We constructed ROC curves for PARP-1 and calculated the area under the curve. The statistical analysis was performed using the SPSS-Windows 15.0 program (SPSS Inc, Chicago, IL, USA). The confidence interval was 95% (p<0.05).

## Results

**Figure 1:**
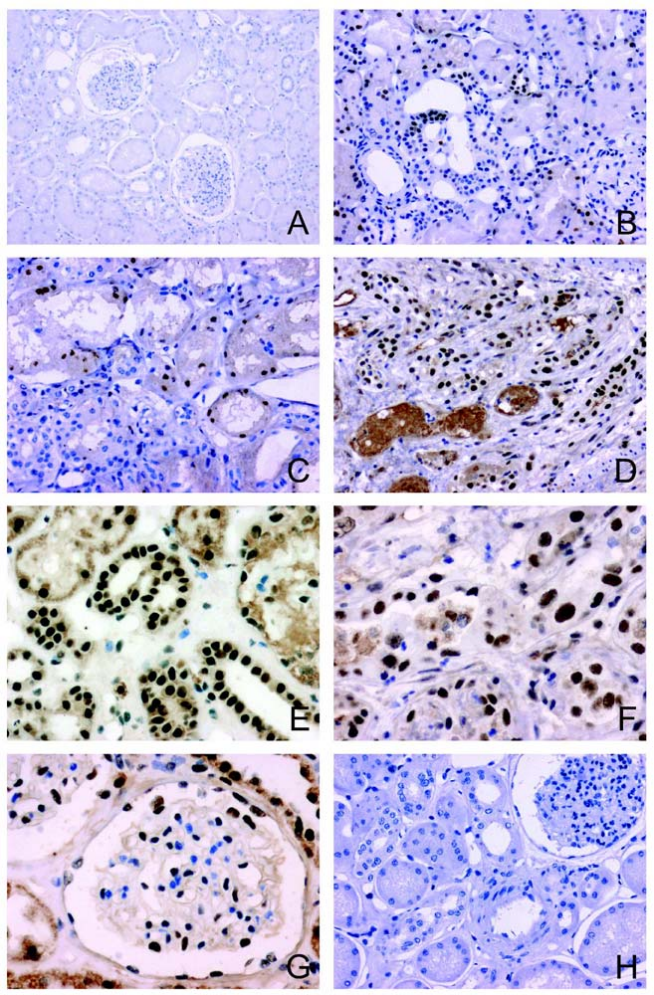
PARP-1 expression in human kidneys using polymer peroxidase-based method. A) Absence of PARP-1 expression in tubular cell nuclei in transplant protocol biopsy of kidney with stable renal function and without ATN (×100). B) Moderate PARP-1 expression in tubular cells of ECD kidney biopsy with ATN (×200). C) Moderate PARP-1 expression in necrotic tubuli of posttransplant kidney biopsy with ATN (×200). D, E, F): Intense PARP-1 expression in various biopsies with severe ATN (D x200, and E, F ×400). G) Glomerular immunostaining in a case of severe ATN. Note nuclear immunostaining in capillary and Bowman's capsule (×400). H) Negative isotype control (x200).

Overall PARP-1 expression in the 326 paraffin-embedded renal allograft biopsies studied was scored as follows: 0 (38.9%); 1 (19.2%); 2 (18.9%); and 3 (23.0%). A score of 0 was only detected in renal biopsies without ATN. The mean age of non-ECDs with ATN was 40.7±10.47. The cold ischemia time was 23.24±5.31 h [range 1–31 h]. The group formed by 98 allograft kidneys with ATN of degree 1 (30.6%), 2 (44.9%), or 3 (24.5%) showed more intense PARP-1 expression (score 2 [45%], score 3 [25%]) at 5–11 days post-transplant versus transplant protocol biopsies with stable renal function (Figure 1). The mean age of ECDs with ATN was 63.63±7.01 yrs and the cold ischemia time was 19.32±4.48 h [range, 8–36 h]. The group formed by 95 pre-transplantation kidney biopsies with ATN of degree 1 (86%), 2 (14%), or 3 (0%) showed a mild nuclear tubular expression of PARP-1.

Table 1 compares the mean values of the four groups of renal biopsies studied and shows the statistical significance of differences found. At time of biopsy, transplanted kidneys with ATN from younger kidney donors (non-ECDs) had higher creatinine serum levels compared with kidneys from ECDs (1.72±0.09 vs. 1.09±0.15, p = 0.0001 Bonferroni test), which had a lower degree of ATN. The group of non-ECD transplanted kidneys with clinical evidence of ATN had longer cold ischemia time and time-to-effective diuresis versus the other groups, including the preimplant ECD kidneys with ATN (one-way ANOVA test, p = 0.0001 for both variables). Degree of ATN was significantly correlated with PARP-1 expression (r coefficient = 0.810, p = 0.0001, Pearson test), with a mean expression of 2.83±0.48 in severe ATN versus 1.53±0.96 in mild cases and 0.33±0.56 in absence of ATN (p = 0.0001, one-way ANOVA test). A significant difference in kidney PARP-1 expression was found between recipients of non-ECD kidneys, which largely showed moderate or intense ATN, and ECD biopsy specimens, which showed only mild or moderate ATN (2.33±0.85 vs. 1.69±0.91, p = 0.0001 Bonferroni test). Degree of ATN was also related to time to recovery of effective diuresis (p = 0.0001, one-way ANOVA test). Cold ischemia time (≤ or >20 h) had a major effect on time to recovery of effective diuresis (4.39±5.3 days vs. 12.41±7.4 days, p = 0.0001, Student's *t*-test), and a duration of >20 h was associated with more than two-fold higher tubular expression of PARP-1 (0.86±0.94 vs. 2.10±1.23, p = 0.0001, Student's *t*-test). Among the 178 kidneys with ≤20 h of cold ischemia, 105 did not have ATN, twenty-three had moderate ATN, and only three had severe ATN (p = 0.0001, chi-square test).

**Table 1:** Comparative data among control kidneys (from expanded-criteria donors [ECD] and patients with stable renal function), preimplant kidneys from ECDs, and transplanted kidneys from patients with ATN.

Variable	ECD without ATN (n = 65)	ECD with ATN (n = 95)	P values Bonferroni test	Non-ECD without ATN (n = 68)†	Non-ECD with ATN (n = 98)	P values Bonferroni test
Age of donor (years)*	58.4±11.9	63.63±7.01	NS	38.70±15.5	38.20±6.33	NS
PARP-1 Score [0–3]*	1.05±0.54	1.66±0.32	P = 0.0001	0.34±0.59	2.33±0.85	P = 0.0001
Age receptor (years)*	50.8±13.2	54.32±11.02	NS	48.4±12.8	40.57±10.47	NS
Cold ischemia time (hours)*	18.39±4.31	19.32±4.48	P = 0.307	19.7±5.09	23.24±5.31	P = 0.112
Anastomosis time (min)	44.4±11.4	44.28±9.54	NS	43.6±12.4	42.06±8.21	NS
Time to efficient diuresis (days)*	2.06±3.33	7.94±5.42	P = 0.0001	0.69±2.67	14.10±4.60	P = 0.0001
N° of hemodialysis*	1.82±2.87	2.63±4.63	P = 0.0001	0.4±0.25	2.06±3.36	P = 0.0001
Creatinine at biopsy (mg/dL)*	1.02±0.10	1.09±0.15	NS	1.32±0.07	1.72±0.09	P = 0.001
Creatinine at one month (mg/dL)*	1.88±0.65	2.35±1.11	P = 0.003	1.51±0.23	3.25±1.37	P = 0.0001
Creatinine at six months (mg/dL)*	1.97±1.05	1.86±0.49	P = 0.55	1.38±0.42	2.94±0.85	P = 0.0001
Creatinine at twelve months (mg/dL)*	1.45±0.50	1.85±0.55	P = 0.001	1.15±0.43	2.90±0.96	P = 0.0001
Creatinine <1.7 (mg/dL) (days)*	22.15±13.8	34.30±27.4	P = 0.001	15.45±9.38	32.3±29.3	P = 0.0001
Cr Clearance at one month*	44.70±19.04	40.52±18.43	NS	55.38±18.5	46.75±12.12	P = 0.001
Cr Clearance at six months*	46.89±18.20	46.05±19.78	NS	59.16±17.8	52.01±19.21	P = 0.01
Cr Clearance at one year*	47.62±13.25	46.75±14.22	NS	59.34±18.7	44.77±19.04	P = 0.005

ECD: Expanded-criteria donor with ATN; ATN: Acute tubular necrosis. Values are expressed as mean ± standard deviation.

* P = 0.0001 one-way ANOVA test and *post hoc* with Bonferroni test.

† Transplant protocol biopsies with stable renal function; NS: Non-significant.

**Figure 2:**
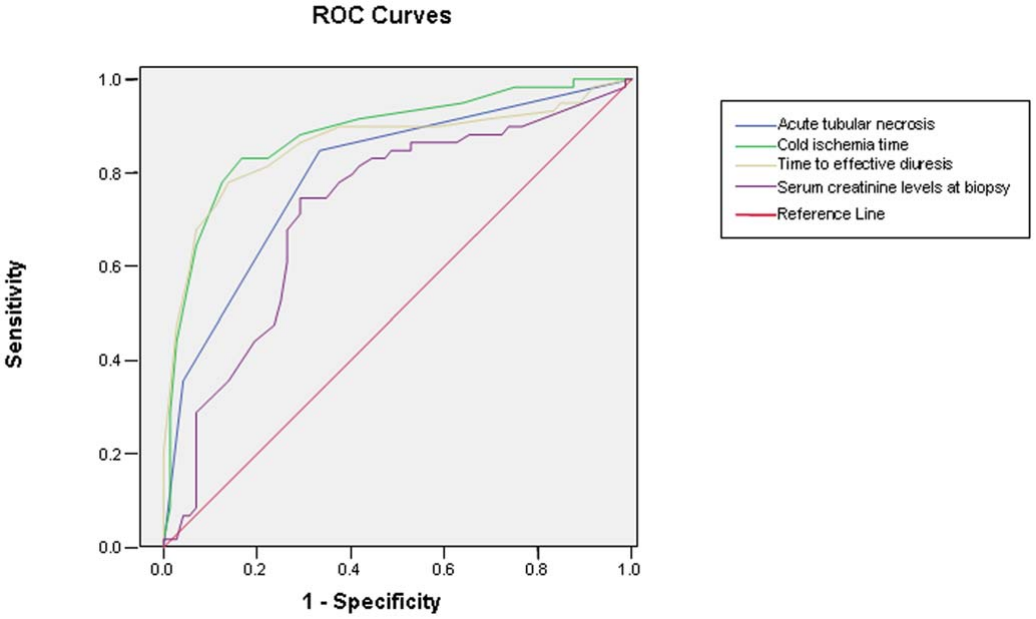
Representation of ROC curves using PARP-1 expression as state variable.

PARP-1 appears to have played an important role in early kidney allogaft function (Table 2), with a statistically significant relationship between its expression and cold ischemia time (r coefficient = 0.603, p = 0.0001, Pearson test), time to effective diuresis (r = 0.770, p = 0.0001, Pearson test), and serum creatinine levels at time of biopsy (r = 0.649) and at three months (r = 0.403, p = 0.0001, Pearson test) but not at six months or one year. Likewise, the degree of ATN showed a significant correlation with the same parameters (cold ischemia time r = 0.456; time to effective diuresis r = 0.696; and creatinine levels at time of biopsy r = 0.520, one month r = 0.455, and six months r = 0.508, p = 0.0001, Pearson test). There was an even greater difference in PARP-1 expression intensity between kidneys from ATN patients who did not reduce serum creatinine levels to below 1.7 mg/dL after transplantation and those from ATN patients who did (creatinine <1.7, PARP-1 1.79±0.62 vs. creatinine >1.7, PARP-1 2.33±1.04, p = 0.0001, Student's *t*-test). Figure 2 and Table 3 represent and summarize the statistical results of the ROC curve analyses, showing the high values of the area under curve for the variables ATN, cold ischemia time, time to effective diuresis, and serum creatinine level at biopsy.

**Table 2:** Clinical variables according to nuclear immunohistochemical expression of PARP-1 in human kidney biopsies.

Variables	PARP-1 negative	PARP-1 positive	P Values*
Age of donor (years)	49.06±17.1	45. 95±14.6	NS
Cold ischemia time (hours)	18.34±5.37	22.12±4.74	P = 0.0001
Time to efficient diuresis (days)	1.65±1.97	10.35±6.75	P = 0.0001
Creatinine at one month (mg/dL)	1.93±2.71	2.71±1.31	P = 0.0001
Creatinine at six months (mg/dL)	1.70±0.76	2.43±1.07	P = 0.0001
Creatinine at twelve months (mg/dL)	1.49±0.78	2.18±1.04	P = 0.0001
Creatinine <1.7 (mg/dL) (days)	5.10±13.55	13.15±18.11	P = 0.0024

Values are expressed as mean ± standard deviation; NS: Non-significant.

* Student's *t-* test.

**Table 3:** Area under the ROC curve, statistical significance and p-values using dichotomous PARP-1 expression in human kidney biopsies as state variable.

Variables	Area	Typ. error	P-values	Superior limit*	Lower limit*
Acute tubular necrosis	0.799	0.039	0.0001	0.721	0.876
Cold ischemia time (hours)	0.882	0.031	0.0001	0.821	0.942
Time to effective diuresis (days)	0.860	0.036	0.0001	0.789	0.930
Serum creatinine levels at biopsy	0.719	0.046	0.0001	0.629	0.809

* 95% Confidence Interval.

**Figure 3:**
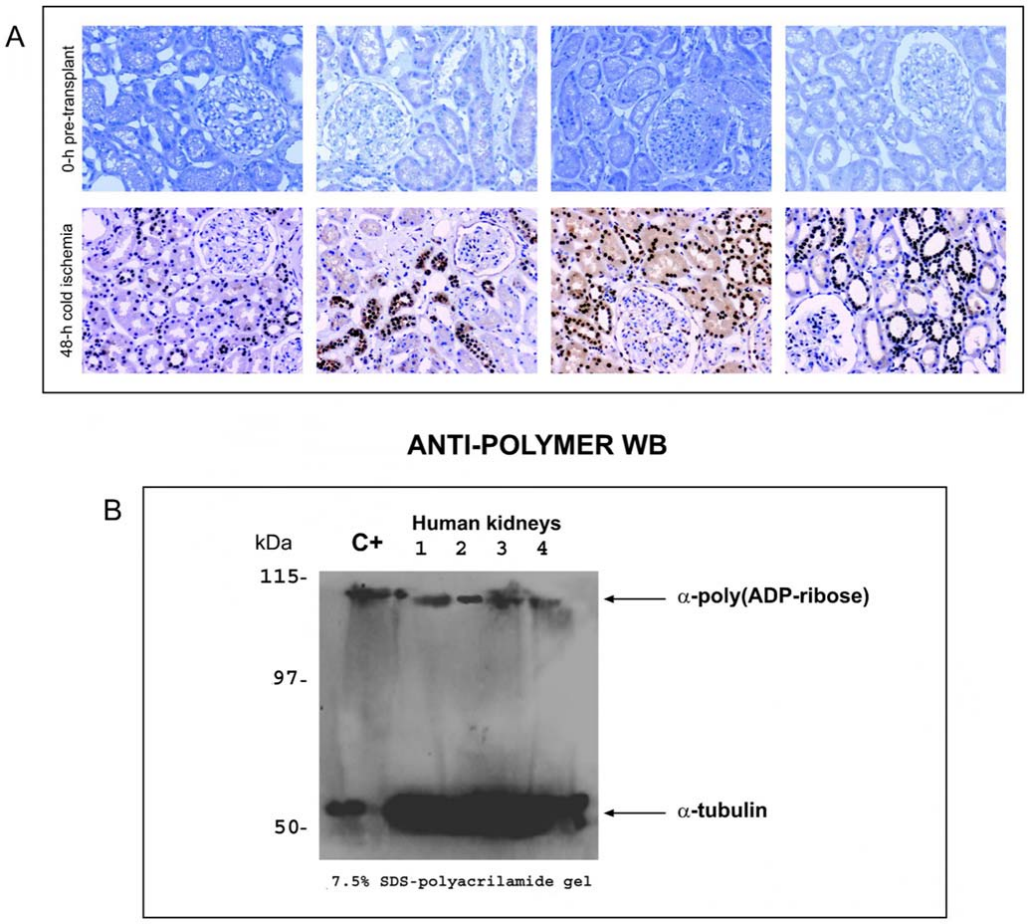
Immunohistochemistry and Western-blot PARP-1 expression in kidneys ruled out for transplantation. A) Representative kidneys ruled out for transplantation but preserved as whole perfused kidneys, immunohistochemistry method revealed a marked increase in PARP-1 nuclear expression between the biopsy at 0 h and the renal cortex after 48 h of cold ischemia in Wisconsin preserved solution. B) Western-blot analysis to detect PARP-1 activation in human kidneys after 48 h cold ischemia (lines 1 to 4). C+ is a pool of Poly-ADP-ribosylated-PARP proteins used as positive control for Western-blot.

In all 20 kidneys ruled out for transplantation but preserved as whole perfused kidneys, immunohistochemistry study revealed a marked increase in PARP-1 expression between the biopsy at 0 h and the renal cortex after 48 h of cold ischemia in Wisconsin preserved solution (Figure 3A), and western blot study showed a mild activation of PARP-1 after the 48 h of cold ischemia (Figure 3B).

**Figure 4:**
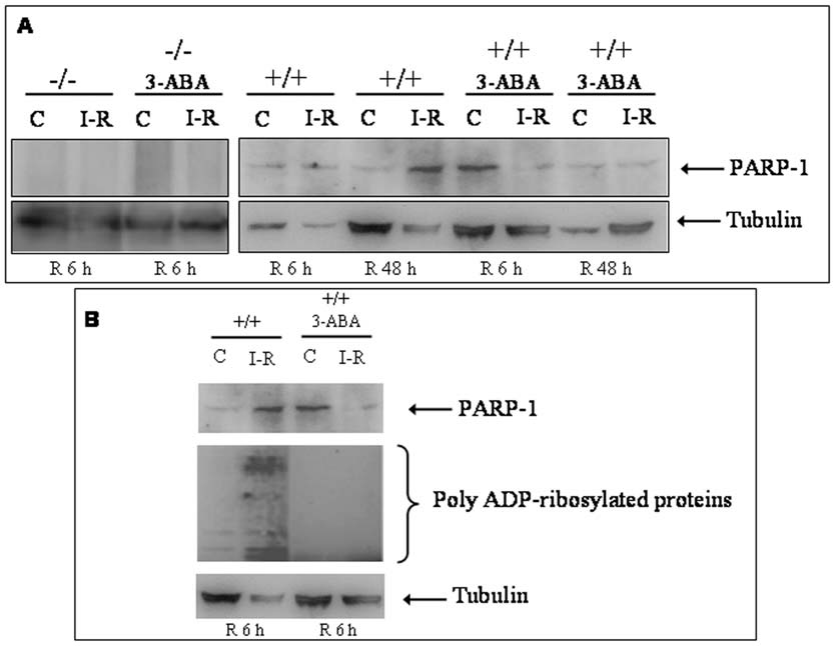
Western-blot analysis of PARP-1 expression in kidney of C57BL/6 mice. A) Presence of PARP-1 expression in kidney of C57BL/6 Parp1^+/+^ mice absence of PARP-1 in knockout mice; and evident increase in PARP-1 expression at 48 h of reperfusion. Note partial inhibition of PARP-1 after inoculation with 3-ABA at 6 h of reperfusion. B) Induction of protein poly(ADP-ribosyl)ation after renal IR and its total inhibition by PARP-1 with 3-ABA. +/+: C57BL/6 wild-type mouse; −/−: C57BL/6 Parp-1 knockout mouse; 3-ABA: 3-aminobenzamide; C: Control; IR: Ischemia-Reperfusion; R: Reperfusion.

In a Parp1 knockout mouse IR model, we used western-blot to demonstrate induction of PARP-1 expression in kidney cortex of C57BL/6 Parp1^+/+^, which was more evident at 48 h (Figure 4A). After 6 h of reperfusion, 3-ABA-treated Parp1^+/+^ mice showed decreased PARP-1 expression and reduced polymer activity (Figure 4B), and no PARP-1 protein was detected by western-blot in Parp1^−/−^ knockout mice (Figure 4A); renal injury appeared to be reduced in these two groups (data not shown).

## Discussion

Increased PARP-1 expression was observed in nuclei of human renal tubular cells after a variable period of cold ischemia and in the same nuclei of patients who developed ATN. PARP-1 expression was inversely correlated with the time to recovery of renal function. PARP-1 induction in tubular cells was previously found in different experimental models of ischemic renal injury [19]–[22], and our group recently demonstrated PARP-1 expression in tubules of aged donors [17]. However, the present study of a large series of kidney transplant patients supports aclose relationship between delayed renal function and tubular nuclear PARP-1 expression. According to our results, PARP-1 protein up-regulation may be a possible and previously unconsidered pathway for delayed graft function (DGF) [23], [24].

A prolonged cold ischemia time is a strong risk factor for DGF, graft loss [4], [6], and long-term changes after kidney transplantation [25]. Donor kidneys inevitably undergo a period of cold ischemia. In our series, the periods of cold ischemia ranged from one to thirty-six hours, with significantly longer times for non-ECDs biopsied for suspicion of ATN (see Table 1). A gradual increase in DGF was significantly correlated with longer cold ischemia time (r coefficient: 0.666, p = 0.01, Pearson test). DGF varied according to cold ischemia time and donor age. It has been reported that ECD transplant performed with a short cold ischemia time (0–8 h) has a DGF rate equivalent to a non-ECD transplant performed with a 20-h cold ischemia time [7].

The present study revealed a significant difference in the intensity of PARP-1 immunohistochemical expression in renal tubular cells between kidneys from transplant protocol biopsies with stable renal function or ECDs without ATN and those from non-ECD transplant patients with ATN or from ECDs with ATN (see Table 1). Results also suggested that a decreased tubular expression of PARP-1 was related to an earlier recovery of renal function. In addition, transplanted kidneys in patients with serum creatinine levels that did not fall below 1.7 mg/dL showed double the intensity of PARP-1 expression. DGF after kidney transplantation may be due to various factors [25], such as the condition of the transplanted kidney and the compliance of the vascular system in the renal graft or recipient. Nevertheless, these findings indicate that the degree of PARP-1 activation may be related to the extent of human renal tubular injury and to renal function, suggesting a role for this enzyme in the pathogenic mechanism of ATN due to IR. In a previous study of patients with ATN, our group found that the kidneys tolerating a long period of cold ischemia had the highest levels of PARP-1 (cold ischemia <24 h, PARP-1  = 1.71±0.62 vs. cold ischemia ≥24 h, PARP-1  = 2.86±0.35). In fact, the lowest PARP-1 expression level (1–9% of tubular nuclei positive) was only observed in kidneys with less than 20 h of cold ischemia (mean, 16.36 h; range, 12–20 h) [26].

The functional capacity of renal tubular cells significantly contributes to an adequate renal function. Hence, measures taken to ameliorate the condition of these cells may also improve the outcome of kidney transplantation [27]. Although the chronic inhibition of PARP activity is likely to be harmful to the cell, it has been proposed that its transient inhibition after IR injury may prevent cell death [15].

Identification of a specific histological biomarker for the early diagnosis of tubular injury in renal biopsies is a current research challenge. Zhang et al. [28] recently used immunohistochemistry to characterize the expression of kidney injury molecule-1 (KIM-1) in renal transplant biopsies, finding a significant correlation between renal functional indices and KIM-1 staining intensity. They suggested that evaluation of KIM-1 staining may serve to optimize the diagnosis of tubular injury in allograft biopsies, similar to our proposal for PARP-1.

In summary, these results suggest a pivotal role for PARP-1 in the ATN of renal transplantation. We propose the immunohistochemical assessment of PARP-1 in kidney allograft biopsies as a risk marker for early detection of a possible delayed renal function.
